# Association Between Common Variable Immunodeficiency and Pulmonary Amyloidosis: Review

**DOI:** 10.3390/jcm15041446

**Published:** 2026-02-12

**Authors:** Cristina Maria Radu, Irena Nedelea, Vlad Andrei Ardelean, Adriana Parau, Milena Adina Man

**Affiliations:** 1 Department of Pneumonology, “Leon Daniello” Clinical Hospital of Pneumophtysiology, 400371 Cluj-Napoca, Romaniamanmilena50@yahoo.com (M.A.M.); 2 Allergy and Immunology Discipline, Iuliu Hațieganu University of Medicine and Pharmacy, 40012 Cluj-Napoca, Romania; 3 Allergy and Clinical Immunology Department, “Octavian Fodor” Regional Institute of Gastroenterology and Hepatology, 400162 Cluj-Napoca, Romania; 4 Department of Internal Medicine, “Octavian Fodor” Regional Institute of Gastroenterology and Hepatology, 400162 Cluj-Napoca, Romania; 5 Department of Medical Sciences-Pulmonology, Faculty of Medicine, University of Medicine and Pharmacy, 8 Victor Babeș Street, 400012 Cluj-Napoca, Romania

**Keywords:** inborn errors of immunity, common variable immunodeficiency (CVID), AA amyloidosis, AL amyloidosis, pulmonary amyloidosis

## Abstract

**Background:** Common variable immunodeficiency (CVID) is the most frequent symptomatic primary antibody deficiency, associated with recurrent infections, immune dysregulation, and non-infectious complications. Amyloidosis is a rare but severe complication with pulmonary involvement being exceptional. **Objective:** The aim of this study was to review reported cases of amyloidosis complicating CVID and present a unique case of pulmonary involvement. **Methods:** A literature research identified observational studies and case reports linking amyloidosis with CVID. Additionally, we describe a patient with CVID complicated by pulmonary and gastrointestinal amyloidosis. **Results:** Fifteen cases were identified, mostly amyloid A (AA) with multiple organ involvement. Only one case of pulmonary amyloidosis was reported. To date, no cases of pulmonary light-chain amyloidosis (AL) have been described in CVID patients without an underlying plasma cell dyscrasia. Our patient initially presented with AA amyloidosis but evolved to systemic AL type with rapid progression and fatal outcome despite therapy. **Conclusions:** Amyloidosis should be considered in CVID patients with atypical symptoms. Accurate amyloid typing is essential as treatment differs between AA and AL types. Early recognition may improve outcomes.

## 1. Introduction

Common variable immunodeficiency (CVID) is an umbrella diagnosis for symptomatic primary antibody deficiencies [1,2,3]. Its clinical phenotype is complex and heterogeneous. CVID disorders are characterized by impaired B cell differentiation, resulting in defective immunoglobulin (Ig) production and a diminished or absent antibody response, which increases the risk of severe recurrent infections [4,5]. Progress in the understanding of the disease indicates that approximately 70% of cases are associated with one or more noninfectious complications, including autoimmunity, enteropathy, polyclonal lymphocytic infiltration (such as splenomegaly and granulomatous infiltration) and malignancy, all stemming from immune dysregulation [2,6]. Consequently, CVID involves defective B cell function, systemic immune activation with abnormal T cell function and dysregulation of other innate immune cells, as well as microbial translocation due to impaired gastrointestinal (GI) barrier function [7]. These abnormal immune responses culminate with a milieu of persistent systemic inflammation [6,8,9,10,11,12,13]. This updated modern scientific view recognizes CVID as a heterogeneous collection of immune dysregulation disorders rather than an infectious complication from low immunoglobulin count [6]. Lifelong treatment with immunoglobulin replacement (IgRT) is essential for managing humoral inborn errors of immunity (IEI) to reduce the risk and severity of infectious complications. However, data on its benefits for noninfectious manifestations remain insufficient [14]. Despite IgRT, patients with noninfectious complications face an 11-fold higher risk of mortality with lung disease being the leading cause of death, followed by lymphoma and liver disease [15].

Lung disease complicates CVID in up to 90% of cases and includes bronchitis (70%), bronchiectasis (30–40%), obstructive ventilatory disease—such as asthma or chronic obstructive pulmonary disease (COPD) (30–50%), granulomatous and lymphocytic interstitial lung disease (GLILD) (up to 20% of cases), and rarely, mucosa-associated lymphoid tissue (MALT) B-cell lymphoma [15,16,17,18,19,20]. Secondary amyloidosis is another possible complication in CVID patients [21].

Amyloidosis is a rare condition wherein proteins are abnormally deposited into extracellular tissue, leading to disruption of existing structures and manifesting in a variety of clinical presentations depending on the organ involved [22]. It can be classified based on quantity, type, and location of these proteins. The most common types of amyloidosis are AL (primary) localized or systemic, systemic amyloid A (AA) and systemic wild-type transthyretin amyloidosis (wt) ATTR. While AL amyloidosis occurs with plasma cell dyscrasias, the AA type accompanies chronic inflammatory diseases such as rheumatic pathologies (familial Mediterranean fever, rheumatoid arthritis, and ankylosing spondylitis), chronic infections (tuberculosis, bronchiectasis, and osteomyelitis), and some malignancies that are observed in patients diagnosed with CVID [23]. Current evidence is largely limited to case reports and small case series, and there is no comprehensive synthesis of the spectrum of amyloidosis subtypes, organ involvement, or patient outcomes. To date, no published cases describing the association of different types of amyloidosis in patients diagnosed with CVID have been identified in the literature. However, the coexistence of different types of amyloidosis in a single patient is a rare but possible occurrence [24].

Given the heterogeneity and limited nature of the existing literature, a scoping review is appropriate to map the available evidence, identify knowledge gaps, and provide an overview of reported cases of amyloidosis occurring as a complication of CVID and also to identify the predominantly affected organs. The main questions were the following. Which types of amyloidosis occur in CVID patients, which organs are most frequently involved, are there cases of coexisting amyloidosis subtypes, and what gaps exist in the current literature? The objectives were to provide an overview of reported cases, identify rare clinically significant events, and highlight areas for future research.

Thus, knowing these data, we want to draw attention to the importance of careful evaluation of CVID patients in order to detect amyloidosis in its early stages in case of persistent symptoms.

## 2. Materials and Methods

This review included case reports and case series published in the PubMed database between 1996 and the present. The search strategy combined the following keywords: (“airway” OR “lung” OR “pulmonary” OR “respiratory”) AND “amyloidosis” AND “CVID”, as well as “amyloidosis” AND “CVID”, applied to all fields.

The primary objective was to identify reported cases of amyloidosis diagnosed in patients with common variable immunodeficiency (CVID) and to explore the potential coexistence of more than one amyloid subtype in the same patient. Given the rarity of this association, the available literature consists predominantly of isolated case reports and small case series, which precludes the application of formal systematic review methodology.

In the first part of the review, we identified case reports and case series describing patients with CVID diagnosed with amyloidosis. Thirteen case reports and one retrospective study reporting CVID complicated by amyloidosis were included.

In the second part of the review, considering the specific features of the index case, we included four case reports and four case series describing the occurrence of more than one amyloid subtype in the same patient. This complementary analysis was intended to provide contextual background and to highlight diagnostic challenges related to overlapping or evolving amyloid phenotypes, rather than to perform an exhaustive review of amyloidosis subtypes.

Studies were considered eligible if individual patient data were available, including age, sex, organ involvement, and amyloid subtype. Missing data were recorded as “not available (N/A)” in the summary tables. This restriction allowed for a focused synthesis and meaningful comparison of clinically relevant cases.

Given that most included studies were case reports or small observational studies, a formal methodological quality assessment using standardized appraisal tools was not applicable. Nevertheless, potential sources of bias, including incomplete clinical descriptions, were considered during data extraction.

The literature search was limited to the PubMed database, which was selected for its comprehensive coverage of biomedical and life sciences journals and its standardized indexing of peer-reviewed clinical reports. Given the rarity of amyloidosis in patients with CVID, this approach was considered sufficient to capture the majority of relevant published cases. However, studies indexed in other databases may have been missed, and this limitation was taken into account in the interpretation of the results.

Accordingly, the findings of this review should be interpreted as descriptive and hypothesis-generating rather than quantitative.

## 3. Results

Using the search strategy described earlier, we identified a total of seventeen cases of amyloidosis associated with CVID, with renal involvement (*n* = 11), GI involvement (*n* = 5), thyroid involvement (*n* = 1), salivary gland involvement (*n* = 1), and pulmonary involvement (*n* = 1). All cases were of the AA subtype (Table 1a,b). In some patients, multiple organ systems were affected simultaneously, reflecting the heterogeneity of clinical presentations. Long-term follow-up data were generally scarce, limiting conclusions about prognosis. Notably, no cases of systemic AL amyloidosis have been described in CVID patients without an associated plasma cell dyscrasia, highlighting the exceptional nature of our observation. These findings emphasize that although uncommon, amyloidosis is a serious complication in CVID with a predominant renal pattern and a potential for systemic progression.

### Representative Case

We report the case of a 71-year-old male with common variable immunodeficiency (CVID), complicated by recurrent respiratory tract infections and bronchiectasis, on long-term immunoglobulin replacement therapy (IgRT) for 10 years. His medical history included hypertension, ascending aortic ectasia, cerebrovascular small-vessel disease, overweight, and impaired fasting glucose. He was a lifelong non-smoker with no relevant occupational exposures.

The patient presented with progressive exertional dyspnea, fatigue, and reduced exercise tolerance over a six-month period, without fever, chest pain, hemoptysis, or systemic inflammatory symptoms.

Pulmonary function tests showed a mixed ventilatory defect with mild restriction and moderate obstruction, while diffusing capacity remained preserved. Pulmonary function test results are presented in Table 2.

**Figure 1:**
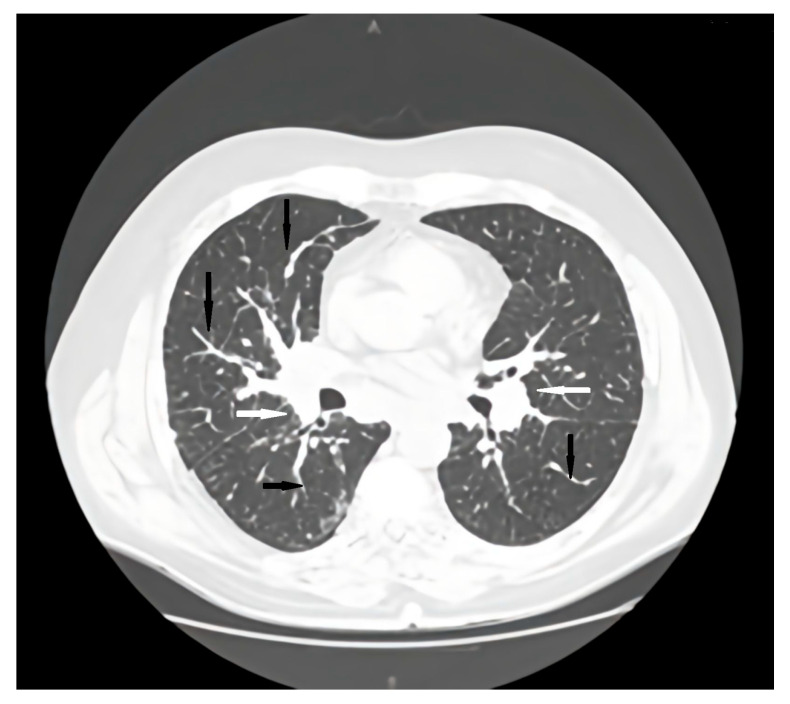
Axial CT scan pulmonary window initial evaluation. A, anterior; P, posterior; black arrows, irregular peribronchovascular thickening; white arrows, adenopathy.

**Figure 2:**
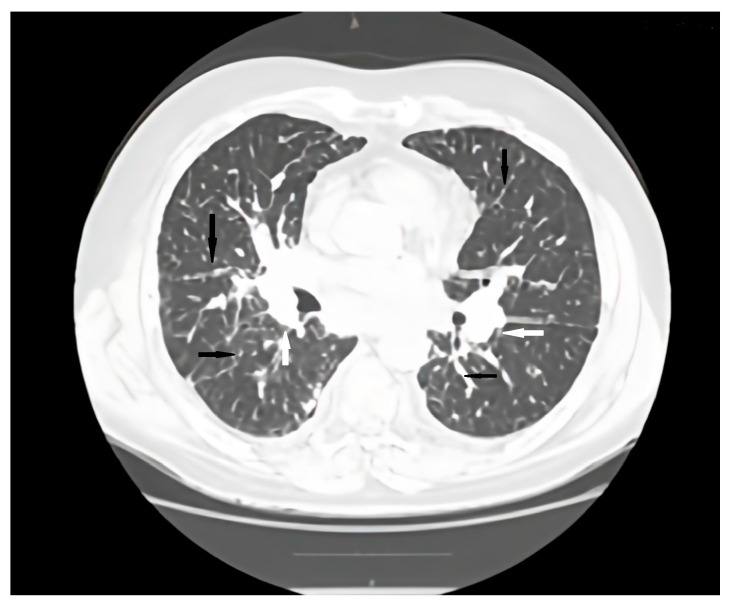
Axial CT scan pulmonary window evaluation after 6 months. A, anterior; P, posterior; black arrows, irregular peribronchovascular thickening; white arrows, adenopathy.

Regular pulmonology follow-up and periodic lung computed tomography (CT) scans showed a stable pattern over the years following the CVID diagnosis, characterized by a bilateral centrilobular micronodular pattern, irregular peribronchovascular thickening, bronchiectasis with mucoid impactations in the lower lobes, alveolar infiltrates, and mediastino-hilar adenopathies. After the onset of respiratory symptoms, a follow-up lung CT revealed similar interstitial findings suggestive of interstitial lung disease along with stable mediastinal-hilar adenopathies (Figure 1 and Figure 2).

At this point, the CT scan was suggestive for an interstitial lung disease (ILD), but its etiology was still unknown. Several differential diagnoses have been considered including GLILD and sarcoidosis. GLILD has been defined as a distinct clinico-radio-pathological ILD occurring in patients with CVID, associated with a lymphocytic infiltrate or granuloma in the lung. The suspicion of pulmonary lymphangitic carcinomatosis was raised in the context of a suspicious gastric lesion identified on non-contrast CT, consisting of gastric wall thickening. Owing to the absence of contrast enhancement, additional details regarding the gastric lesion could not be assessed.

Bronchoscopy demonstrated hyperemic bronchial mucosa and two small translucent nodular lesions in the intermediate bronchus. Histopathological examination of bronchial biopsies revealed Congo red–positive amyloid deposits in a nodular pattern, confirming nodular pulmonary amyloidosis; however, amyloid subtyping by immunohistochemistry or immunofluorescence was not performed at that time. Bronchoalveolar lavage showed no evidence of malignancy, infection, or lymphocytic alveolitis. Cultures did not reveal any pathogens including common germs Aspergillus and Mycobacterium tuberculosis.

**Figure 3:**
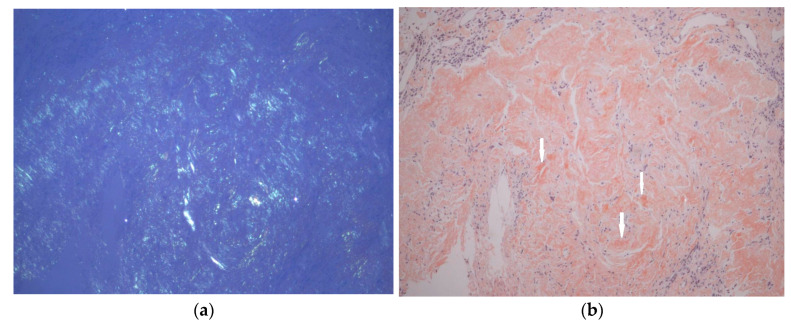
Gastric biopsy revealing amyloid deposits with apple-green birefringence under Congo red staining. Images were acquired using a Leica DFC450C microscope (Leica Microsystems GmbH, Wetzlar, Germany), 200× objective, the scale bar represents 50 μm. (**a**), polarized light; (**b**), Congo red, white arrows, amyloid deposits.

Given the limitations of non-contrast imaging in characterizing gastric lesions, upper gastrointestinal endoscopy was subsequently performed and revealed diffusely infiltrated gastric mucosa with nodular and ulcerative lesions. Gastric and duodenal biopsies confirmed amyloid deposition, ruling out malignancy (Figure 3). The patient was asymptomatic from a gastrointestinal standpoint.

Whole-exome sequencing (WES) for a primary immunodeficiency panel of 575 genes was performed with support from the Jeffrey Modell Foundation (JMF). The results confirmed carrier status for thrombocytopenia-absent radius syndrome and identified several variants of uncertain significance.

Initial laboratory investigations were largely unremarkable, including inflammatory markers, renal and liver function tests, cardiac biomarkers, autoimmune serology, and tumor markers. Detailed laboratory findings are summarized in Table 3. Serum amyloid A protein was mildly elevated. Serum protein electrophoresis with immunofixation showed no monoclonal bands, and genetic testing excluded hereditary transthyretin amyloidosis.

Based on the clinical presentation, history of CVID, CT findings, histopathological results, and complementary investigations available at the initial evaluation, the working diagnosis was secondary (AA) amyloidosis with pulmonary and gastric involvement, most likely related to recurrent respiratory infections. In addition to regular IgRT (600 mg/body weight/month), treatment with corticosteroids and azithromycin prophylaxis led to transient symptomatic improvement.

At three-month follow-up, the patient experienced rapid clinical deterioration with severe dyspnea, weight loss, early satiety, atrial fibrillation, and signs of autonomic dysfunction. Laboratory evaluation revealed mild anemia, inflammatory syndrome, hypoproteinemia, mild proteinuria, and elevated fecal calprotectin.

**Figure 4:**
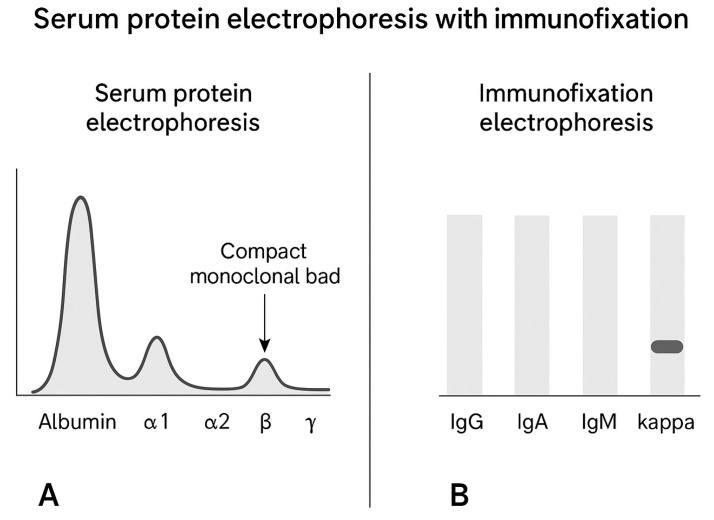
Serum protein electrophoresis with immunofixation. (**A**), serum protein electrophoresis (SPEP) showing a compact monoclonal band in the γ region; (**B**), immunofixation electrophoresis (IFE) demonstrating a strong monoclonal band in the kappa light chain lane, confirming the presence of a monoclonal kappa component.

Subsequent hematological evaluation identified additional manifestations suggestive of systemic amyloidosis, including cardiac involvement (orthostatic hypotension), peripheral neuropathy, macroglossia, and sicca syndrome. These findings, together with abnormal serum free light chain levels and a deranged kappa:lambda ratio, raised suspicion for AL amyloidosis. An extended workup was performed including serum protein electrophoresis with immunofixation where a compact band in the Ig area for kappa light chain was detected (Figure 4). Serum immunofixation showed an elevated free kappa light chain and a normal free lambda light chain, with kappa:lambda ratio, 196.2.

Bone marrow biopsy done to rule out multiple myeloma or other lymphoproliferative disorders showed no signs of plasma cell dyscrasia These findings, together with abnormal serum free light chain levels and a deranged kappa:lambda ratio, raised suspicion for AL amyloidosis.

Treatment with daratumumab and cyclophosphamide was initiated.

Despite therapy, the patient developed sepsis and acute respiratory failure and died one year after the diagnosis of pulmonary amyloidosis due to multiorgan failure secondary to *Candida albicans* fungemia. A summary of the clinical course is provided in Table 4.

A post mortem examination was not performed, as consent for autopsy was declined by the family in the context of a previously established diagnosis of systemic amyloidosis. As a result, the extent of organ involvement and amyloid subtyping could not be assessed, and the potential coexistence of AA and AL amyloidosis remains unconfirmed.

Although secondary AA amyloidosis is most commonly reported in patients with CVID, the overlap in clinical presentation raises the theoretical possibility of coexistence of different amyloid subtypes. However, immunohistochemical amyloid typing was not available in this case. Consequently, the presence of concomitant AA and AL amyloidosis could not be confirmed.

After reviewing the literature, we identified four case reports and four case series describing the co-existence of two types of amyloidosis (Table 5). Twenty-two cases were reported with ages ranging from 31 to 90 years and a predominance of male patients. The underlying conditions were variable, including monoclonal gammopathy of unknown significance (MGUS), chronic kidney disease, lymphoplasmacytic lymphoma, smoldering myeloma, and heart failure, although several patients had no reported comorbidities. The most frequently observed combinations involved AL amyloidosis coexisting with ATTR (wild-type or hereditary) or SAA amyloidosis. Cardiac involvement was common, either as part of systemic amyloidosis or in combination with other organ involvement such as the kidneys, liver, duodenum, or bone marrow [24,39,40,41,42,43,44,45].

The temporal relationship between the diagnoses of the two amyloidosis subtypes varied widely, ranging from simultaneous detection to intervals of several years (up to 21 years), suggesting both synchronous and metachronous presentations. These cases highlight the heterogeneity in clinical presentation, organ involvement, and underlying conditions among patients with coexisting amyloidosis, emphasizing the importance of comprehensive evaluation and long-term monitoring.

Overall, the results emphasize that the current evidence is largely based on case reports and small case series, with limited systematic data. This highlights the need for larger cohort studies to better characterize the prevalence, risk factors, and long-term outcomes of amyloidosis in CVID.

## 4. Discussion

Common variable immunodeficiency (CVID) represents an umbrella term for symptomatic primary antibody deficiencies, affecting approximately 1 in 25,000 individuals, with a higher prevalence in Northern Europe [1]. No clear predilection for race or sex has been identified. Although CVID can present at any age, most cases manifest during adulthood, with a peak incidence between the second and fourth decades of life [1,2,3].

In the present case, the diagnosis of CVID was established based on severe hypogammaglobulinemia, with markedly reduced serum levels of IgG (427 mg/dL; age-adjusted lower limit 670 mg/dL), IgM (14.12 mg/dL), and IgA (21.20 mg/dL), in combination with recurrent upper and lower respiratory tract infections (three to four episodes of pneumonia per year). These infections had begun approximately six years prior to diagnosis and were initially managed by the family physician and other specialists. A detailed clinical history, however, revealed recurrent respiratory infections dating back to childhood. Despite this, the diagnosis of CVID was made late, at the age of 62, representing a diagnostic delay of more than two decades. By the time immunological evaluation was performed, the patient had already developed bronchiectasis, complicating disease management and negatively affecting quality of life. Consequently, initiation of appropriate immunoglobulin replacement therapy (IgRT) was significantly delayed. It is well established that delayed diagnosis and delayed initiation of IgRT in CVID are associated with increased morbidity and mortality [46]. Bronchiectasis remains one of the most frequent complications in patients with inborn errors of immunity (IEIs) [46]. In such cases, the primary therapeutic goals are normalization of serum IgG levels and reduction of infectious complications [47]. Prophylactic and therapeutic antibiotics, as well as vaccination with inactivated antigens, are recommended in these patients, including those with protein-losing conditions and pregnant individuals [48].

Although IgG trough levels were initially within the normal range, adequate control could not be maintained in later months despite dose escalation of IgRT. This was attributed to protein loss secondary to systemic amyloidosis with renal, gastrointestinal, and likely hepatic involvement.

Amyloid subtyping by immunohistochemistry or immunofluorescence (AA protein, kappa and lambda light chains) was not performed on tissue samples, as histopathological processing was carried out in another institution and detailed amyloid typing was unavailable. Therefore, definitive histological differentiation between AA and AL amyloidosis could not be established. Currently, there is no standardized approach regarding the timing or methodology for amyloidosis evaluation in patients with CVID, particularly in cases with pulmonary involvement.

Further investigations, including serum protein electrophoresis with immunofixation, initially showed no monoclonal IgG, IgA, or IgM bands and no monoclonal kappa or lambda light chains, while serum amyloid A levels were elevated, and genetic testing excluded ATTR amyloidosis. These findings supported the initial interpretation of secondary (AA) amyloidosis associated with CVID, in line with current guidelines recognizing primary immunodeficiency among potential causes of AA amyloidosis.

Over a six-month period, the patient developed additional clinical and laboratory features suggestive of systemic AL amyloidosis, including cardiac involvement, peripheral neuropathy, macroglossia, and sicca syndrome. Repeat hematological evaluation identified a compact kappa light chain band on serum immunofixation, with marked elevation of free kappa light chains and a severely deranged kappa:lambda ratio, while bone marrow biopsy did not reveal plasma cell dyscrasia.

Although these findings raised suspicion for systemic AL amyloidosis, the absence of tissue-based amyloid subtyping precluded definitive differentiation between amyloid subtypes or confirmation of their coexistence. Rather than establishing a diagnosis of coexistent AA and AL amyloidosis, this case illustrates the diagnostic complexity and evolving phenotype of amyloidosis in CVID.

Sequential development of different amyloid-related clinical features cannot be excluded; however, definitive conclusions require tissue-based amyloid subtyping. A major limitation of this case is the absence of immunohistochemical amyloid typing, which precluded definitive characterization of the amyloid subtype and the extent of organ involvement. Consequently, any discussion regarding coexistence of different amyloid types should be interpreted with caution.

The absence of initial immunohistochemical or immunofluorescence staining represents a well-recognized diagnostic pitfall in amyloidosis, as reliance on clinical features and non-specific histopathology may lead to misclassification of the amyloid subtype or delayed diagnosis. This is particularly relevant in CVID, in which overlapping inflammatory and hematologic features can further complicate amyloid typing.

Beyond this individual case, the broader context of CVID and its complications provides key insights into disease heterogeneity, mechanisms of immune dysregulation, and the pathophysiology of amyloidosis in primary immunodeficiencies.

According to the current scientific view, CVID is a heterogeneous collection of disorders characterized by immune dysregulation rather than an infectious complication of low immunoglobulin counts [6]. The two primary features of the disease are impaired B cell differentiation with defective immunoglobulin production and a reduced or absent antibody response, along with immune dysregulation of both innate and adaptive cells. These factors are likely interconnected through abnormal microbiota and microbial translocation due to impaired GI barrier function. Together, these features contribute to a milieu of persistent systemic inflammation [6,8,9,10,11,12,13]. It is now accepted that not only B cells are affected but also certain T cell subsets, dendritic cells, monocytes, innate immune lymphoid cells, and natural killer T cells (NKT cells) [14]. Several defects in innate and adaptive immune responses have been shown to play a role in the development of this wide range of infectious and noninfectious complications, although the exact molecular defects leading to disease remain a topic of study and interpretation [14].

CVID disorders present a unique challenge, as they fall under an umbrella diagnosis currently governed by non-universal diagnostic criteria (Table 6). Differential diagnoses of hypogammaglobulinemia are summarized in Table 7.

In recent years, several monogenic disorders leading to the CVID phenotype have been identified in less than 20% of patients in nonconsanguineous cohorts and in approximately 70% of patients in consanguineous cohorts [48]. State of the art diagnosis of IEI involves genetic testing of patients, with next generation sequencing being the gold standard [50].

Secondary amyloidosis, resulting from ongoing inflammation due to recurrent infections and autoimmunity, is an expected clinical presentation in patients with CVID. There are few cases in the literature describing the coexistence of two forms of amyloidosis [23,40,41,43,44,45,51], but no case reported this association in patients with CVID. The low prevalence can be attributed to several factors, starting with the non-specific clinical signs, which can easily be mistaken for other conditions. Additionally, some CVID patients may be at higher risk of developing amyloidosis due to chronic and recurrent infections, often resulting from a delayed CVID diagnosis or patient negligence.

Considering that ATTR amyloidosis was excluded following genetic testing, the discussion focused on the two remaining possible forms, AL and AA.

AL (primary) amyloidosis showed a male predominance with a median age at diagnosis of 64 years and a male-to-female ratio of approximately 3:2 [52]. The estimated incidence in Western Europe and the United States is 6–10 cases per 100,000 individuals annually [53], although precise figures remain unknown. Early mortality remains high, reflecting persistent diagnostic delays, with nearly 20% of patients dying within six months of diagnosis—a statistic that has not improved over the past four decades despite advances in therapy [43,53].

Secondary amyloidosis (AA) in CVID typically arises from chronic inflammation due to recurrent infections or autoimmune processes. Clinical manifestations vary depending on the organs involved with up to 70% of patients presenting with multi-organ involvement at diagnosis [54]. The heart (71%), kidneys (58%), and nervous system (23%) are the most frequently affected organs, followed by the liver (16%) and, less commonly, the GI tract, joints, thyroid, gums, and lungs [53,55].

### Pulmonary Involvement in Amyloidosis

Pulmonary amyloidosis, although rare, is most often associated with AL rather than AA amyloidosis and may occur in localized or systemic forms [56]. Autopsy studies indicate that pulmonary involvement is present in 88% of systemic amyloidosis cases, with a poor median survival of 16 months [55].

Three main patterns of pulmonary amyloidosis have been described: tracheobronchial, nodular parenchymal, and diffuse parenchymal, with the latter being the least common (approximately 3% of pulmonary amyloidosis cases) and often associated with multiple myeloma and poor prognosis [22]. Diffuse alveolar-septal amyloidosis is characterized by amyloid deposits in alveolar septa and vessel walls and is usually linked to systemic AL amyloidosis, although cases caused by systemic AA or hereditary ATTR amyloidosis have also been reported [22].

Bronchoscopy with biopsy (transbronchial lung biopsy or cryobiopsy) is considered a diagnostic gold standard for certain diffuse alveolar-septal or tracheobronchial forms, especially when imaging and non-invasive tests are inconclusive [57]. Histopathology typically reveals amorphous eosinophilic amyloid deposits in alveolar septa, particularly around capillaries, demonstrating apple-green birefringence with Congo Red staining under polarized light [22].

The differential diagnosis of pulmonary amyloidosis includes metastatic disease, congestive heart failure, miliary tuberculosis, sarcoidosis, silicosis, hypersensitivity pneumonitis, and other interstitial lung diseases [58]. In CVID, GLILD represents a distinct entity characterized by lymphocytic infiltration or granuloma formation in the lungs [22].

Gastrointestinal involvement in amyloidosis, despite growing awareness in recent years [59], is frequently overlooked or diagnosed late due to its nonspecific clinical presentation and heterogeneous endoscopic appearance, particularly in patients without previously established systemic disease. This diagnostic challenge may result in significant morbidity, including gastrointestinal bleeding, malabsorption, protein-losing enteropathy, and weight loss, emphasizing the need for high clinical suspicion in appropriate settings [60,61,62].

Primary AL amyloidosis frequently affects the gastrointestinal tract, often alongside cardiac and renal involvement, whereas other subtypes—including AA amyloidosis, hereditary or wild-type ATTR amyloidosis, β2-microglobulin–related amyloidosis, and rarer forms such as AGel—may also involve the gastrointestinal system to varying degrees [59,60]. Awareness of subtype-specific organ tropism is essential for guiding diagnostic evaluation and management.

Gastrointestinal amyloidosis may occur as part of systemic disease or, less commonly, as a localized process. Amyloid deposition can involve any segment of the gastrointestinal tract and may affect the mucosa, submucosa, muscularis propria, or vasculature, leading to a wide spectrum of clinical manifestations, including abdominal pain, diarrhea, weight loss, and gastrointestinal bleeding [60,63]. These manifestations may result from infiltrative involvement causing mucosal fragility or vascular compromise, as well as from autonomic dysfunction due to amyloid infiltration of the enteric nervous system [62]. Importantly, gastrointestinal involvement may be clinically significant even in the absence of overt endoscopic abnormalities.

Endoscopic findings are heterogeneous and often nonspecific, ranging from mucosal friability, erosions, ulcerations, and thickened folds to nodular or polypoid lesions and white plaques, depending on the depth and distribution of amyloid deposition [63]. Diagnostic yield varies by anatomical site, with higher detection rates reported in the small intestine, highlighting the importance of targeted biopsy sampling. Histopathological confirmation using Congo Red staining with apple-green birefringence under polarized light remains essential [59,60,63].

In this context, the present case further illustrates the diagnostic challenge of gastrointestinal amyloidosis, emphasizing that gastrointestinal involvement may represent an initially unsuspected manifestation of systemic disease. The nonspecific clinical and endoscopic findings observed in our patient are consistent with previously reported data and reinforce the importance of histopathological confirmation and heightened clinical awareness to avoid delayed diagnosis [60,61,62].

Accurate amyloid protein typing is critical for guiding treatment. Clinical presentations range from incidental asymptomatic findings on biopsy to severe multi-organ dysfunction [24]. AL amyloidosis predominantly affects the kidneys, heart, and liver, whereas AA commonly involves the kidneys, liver, and intestines [44].

Therapeutic strategies differ by amyloid subtype. Systemic AL amyloidosis is primarily managed with chemotherapy targeting plasma cell clones, aiming to eradicate the pathogenic clone [64], while AA amyloidosis treatment focuses on controlling the underlying inflammatory process to reduce SAA levels [65]. Diagnosis of AA amyloidosis requires tissue confirmation via biopsy—abdominal fat aspirate is preferred, though biopsy of a clinically affected organ is acceptable [65]. Immunofluorescence or immunohistochemical staining is then performed to differentiate AA from AL amyloidosis, with monospecific anti-AA protein antibodies confirming AA amyloidosis and the absence of kappa or lambda light chain staining supporting AL amyloidosis [66].

The findings of this review indicate that the current evidence on amyloidosis in CVID is predominantly derived from case reports and small case series, with no large prospective studies available. Within the literature, AA amyloidosis was the most frequently reported subtype among CVID patients, whereas AL amyloidosis was more commonly observed in cases where two types of amyloidosis coexisted. Renal involvement emerged as the most consistent clinical manifestation in CVID patients, while cardiac amyloidosis predominated in cases of coexisting amyloid subtypes.

These results directly address the review objectives by delineating the spectrum of amyloidosis types and organ involvement in CVID while highlighting critical gaps in the existing evidence. For clinicians, the findings emphasize the importance of vigilant monitoring for amyloidosis in CVID patients. For researchers, they underscore the need for systematic studies and larger cohorts to better characterize the epidemiology, clinical course, and outcomes of amyloidosis in this population.

This review has several limitations. First, the number of published cases of amyloidosis in CVID patients is very limited, and most evidence comes from isolated case reports or small case series, making it difficult to draw firm conclusions and increasing the risk of publication bias, as unusual or severe cases are more likely to be reported. Additionally, long-term follow-up data are scarce.

From a methodological perspective, the search strategy was limited to PubMed, and studies indexed in other databases such as Embase [67], Scopus [68], or Web of Science [69] may have been missed. Moreover, a formal review protocol was not registered, which may reduce transparency and reproducibility.

These limitations highlight the need for more systematic reporting of amyloidosis in CVID and for multicenter studies using standardized diagnostic and reporting approaches. Despite these limitations, the synthesis emphasizes the need for heightened clinical vigilance for amyloidosis in CVID patients presenting with atypical organ dysfunction, for routine consideration of amyloid typing (including immunohistochemistry or immunofluorescence on tissue and appropriate hematologic evaluation), and for multicenter studies to better define epidemiology and optimal diagnostic-therapeutic pathways.

## 5. Conclusions

### 5.1. Clinical Implications

Inborn errors of immunity frequently remain undiagnosed, and diagnostic delays increase the risk of severe complications. Amyloidosis of the lower respiratory tract is rare and often overlooked, yet it may pose a significant diagnostic and therapeutic challenge in both systemic and organ-limited forms. Although reported cases are few, amyloidosis represents a clinically important complication in patients with common variable immunodeficiency and may be underrecognized. Clinicians should therefore maintain a high index of suspicion for amyloidosis in CVID patients presenting with atypical or progressive organ involvement.

Accurate amyloid subtyping is essential, as secondary AA amyloidosis is most commonly associated with CVID, but primary AL amyloidosis should also be considered. Identification of the amyloidogenic protein is critical, given the substantial differences in management strategies among amyloidosis subtypes.

### 5.2. Implications for Future Research

Given the rarity of amyloidosis in CVID and the predominance of case-based evidence, further multicenter studies and systematic data collection are needed to better define prevalence, clinical phenotypes, and optimal diagnostic approaches. Increased awareness and standardized diagnostic pathways may improve early detection and outcomes in this vulnerable patient population.

## Figures and Tables

**Table 1:** (**a**) Literature review of cases describing amyloidosis associated with CVID [25,26,27,28,29,30,31,32,33,34,35,36,37,38,39]. (**b**) Organ distribution of amyloidosis.

(**a**)					
**Year of Publication**	**Age**	**Gender**	**Underlying Chronic Condition**	**Organ Involvement**	**Type of Amyloidosis**
2015	40 years	Male	CVID	Renal	AA-amyloidosis
2010	29 years	Female	CVID	Renal	AA-amyloidosis
2009	37 years	Male	CVID	Thyroid	AA-amyloidosis
2014	20 years	Female	CVID	GI tract	AA-amyloidosis
2005	28 years	Male	CVID	GI tract	AA-amyloidosis
2012	24 years	Female	CVID	Renal and GI tract	AA-amyloidosis
2011	66 years	Male	CVID	Renal	AA-amyloidosis
2022	47 years	Female	CVID	Renal	AA-amyloidosis
2015	27 years	Male	CVID	Renal	AA-amyloidosis
2015	24 years	Male	CVID	Renal and pulmonary	AA-amyloidosis
2015	66 years	Female	CVID	GI tract	AA-amyloidosis
1996	49 years	Female	CVID	Renal	AA-amyloidosis
2010	29 years	Male	CVID	GI tract	AA-amyloidosis
2010	50 years	Male	CVID	Renal	AA-amyloidosis
2020	Not available (N/A)	N/A	CVID	Renal	AA-amyloidosis
2020	26 years	N/A	CVID	Salivary gland	AA-amyloidosis
	68 years	Female	CVID	Renal	AA-amyloidosis
(**b**)					
**Organ Involvement**		**Reported Cases (*n*)**		**Amyloid Subtype(s)**	
Lung		1		AA-amyloidosis	
Gastrointestinal tract		5		AA-amyloidosis	
Kidney		11		AA-amyloidosis	
Thyroid		1		AA-amyloidosis	
Salivary gland		1		AA-amyloidosis	
Multiorgan involvement		2		AA-amyloidosis	

No formal critical appraisal of the included sources of evidence was undertaken as the purpose of this review was to provide an overview of the existing literature rather than assess the methodological quality of individual studies.

**Table 2:** Pulmonary function tests at presentation.

Parameter	Predicted (%)	Absolute Value
FVC	61%	2.36 L
FEV_1_	57%	1.69 L
FEV_1_/FVC ratio	0.67	0.74
DLCO	79%	7.02 L
KCO	102%	1.29 L
Alveolar volume (AV)	80%	5.42 L

**Table 3:** Laboratory investigations at initial evaluation and during follow-up.

Parameter	Reference Range	Initial Evaluation	Follow-Up
Hemoglobin (g/dL)	14–18	14.4	13.9
White blood cells (×10^3^/µL)	4–10	4.48	6.96
Platelets (×10^3^/µL)	150–450	348	256
C-reactive protein (mg/L)	<5	6	7.24
Total serum protein (g/dL)	6.6–8.3	6.1	5.7
Albumin (g/dL)	3.5–5.0	4.8	4.2
AST (U/L)	<40	16	37
ALT (U/L)	<40	17	122
Creatinine (mg/dL)	0.7–1.2	0.82	0.65
Urea (mg/dL)	16–48	45	63
NT-proBNP (pg/mL)	<300	73.2	Not reported
IgG (mg/dL)	700–1600	348	420
IgA (mg/dL)	70–400	11	261
IgM (mg/dL)	40–230	9.45	187
B cells (cells/µL)	78–899	20	Not reported
Serum amyloid A (mg/L)	<10	16	Not reported
Free kappa light chains (mg/dL)	3.3–19.4	Not reported	1057.44
Free lambda light chains (mg/dL)	5.7–26.3	Not reported	5.5
Kappa:lambda ratio	0.26–1.65	Not reported	196.2
Proteinuria (mg/24 h)	<150	Not reported	193.5
Fecal calprotectin (µg/g)	<50	Not reported	354

**Table 4:** Timeline of clinical course.

Timepoint	Key Findings
Baseline	CVID on IgRT; progressive dyspnea; ILD on CT; pulmonary and gastric amyloid confirmed histologically
Initial diagnosis	Secondary (AA) amyloidosis suspected; immunosuppressive therapy initiated
3 months	Worsening dyspnea, atrial fibrillation, early satiety, weight loss
6 months	Multiorgan involvement (cardiac, neurologic, soft tissue); abnormal serum free light chains
Hematologic reassessment	Compact kappa band on immunofixation; AL amyloidosis suspected
Treatment	Daratumumab + cyclophosphamide
Outcome	Death due to sepsis and multiorgan failure

**Table 5:** Cases presenting combined types of amyloidosis in a single patient [24,39,40,41,42,43,44,45].

Year of Publication	Authors	Age (Years)	Gender	Underlying Chronic Condition	Type of Amyloidosis	Period of Time Between Diagnosis
2014	Mahmood et al.	81	male	Not available (N/A)	Cardiac wild-type transthyretin amyloidosis (wtATTR) and AL amyloidosis	At the same time
2018	Jhaveri et al.	69	male	Lymphoplasmacytic lymphoma	Renal AL amyloidosis and cardiac wtATTR amyloidosis	6 years
		63	male	N/A	Renal and liver AL amyloidosis and cardiac wtATTR amyloidosis	21 years
N/A	Papa et al.	31	female	Renal failure	Renal AA amyloidosis and duodenum AL amyloidosis	13 years
2019	Sidiqi et al.	86	female	MGUS	Gastric wtATTR and SAA from fad pad biospy	4 months
		74	male	MGUS	Cardiac ATTR amyloidosis from fat pad biopsy	At the same time
		84	male	N/A	Kidney AL amyloidosis and bone marrow ATTR amyloidosis	2 months
		90	male	N/A	Heart ATTR amyloidosis and duodenum AL amyloidosis	At the same time
		59	male	N/A	Bone marrow AL and ATTR amyloidosis	At the same time
		59	male	lymphoma	Bone marrow Al and ATTR amyloidosis	At the same time
		79	male	N/A	Cardiac AL and ATTR amyloidosis and Renal AL amyloidosis	4 months
		70	male	N/A	Bone marrow AL amyloidosis and ATTR amyloidosis	130 months
		66	male	N/A	AL amyloidosis from fat pad biopsy and cardiac AL and ATTR amyloidosis	1 month
2019	Martini et al.	73	male	MGUS	wtATTR and SAA from fat pad biopsy	At the same time
		70	male	Chronic kidney disease (CKD) G3, chronic heart failure (CHF) NYHA II	Cardiac wtATTR and AL amyloidosis	At the same time
		76	male	CKD G3, MGUS, hypertension	AL and SAA amyloidosis from fat pad biopsy	At the same time
		80	male	CKD G2, CHF NYHA II, smouldering myeloma	AL and wtATTR amyloidosis from fat pad biposy	At the same time
2022	Yu et al.	83	male	N/A	Cardiac AL and ATTR amyloidosis	At the same time
2024	Eda et al.	78	female	MGUS	Bone marrow ATTR amyloidosis and tongue ATTR and AL amyloidosis	5 months
2024	Gami et al.	83	male	CHF, MM	Cardiac AL and wtATTR amyloidosis	At the same time
		90	male	CHF	Cardiac AL and wtATTR amyloidosis	At the same time
		85	male	CKD, CHF	Cardiac AL and hATTR amyloidosis	At the same time

**Table 6:** Comparison of diagnostic criteria for common variable immunodeficiency disorder.

ICON (International Consensus Document) Guide	ESID (European Society of Immune Deficiencies) [49]
A decreased serum level of IgG, measured on two separate occasions at least 3 weeks apart. Low serum levels of IgM or IgA. Altered response to vaccination. Age over 4 years. Exclusion of other causes of secondary hypogammaglobulinemia (Table 5).	The reduction of IgA is a mandatory criterion. A reduced level of switched memory B cells (<70% of the value predicted for the patient’s age) can replace the measurement of antibody titers at vaccination. No profound T lymphocyte deficiency. Clinical manifestations of the disease include increased susceptibility to infections, signs and symptoms of autoimmune disease, granulomatous disease, polyclonal lymphoproliferation, or a known family history of humoral immunodeficiency. The treatment consists of lifelong IgRT to diminish the risk of future infections [47].

**Table 7:** Differential diagnosis of hypogammaglobulinemia.

Drug-Induced	Gene Defects	Infectious Diseases	Malignancies, Other Autoimmune Diseases
Antimalarials Captopril Carbamazepine Corticosteroids Mycophenolate mofetil Cyclophosphamide Gold salts Phenytoin Penicillamine Sulfasalazine Methotrexate Chemotherapy drugs Anti CD-20, Imatinib, Atacicept Chlorpromazine	Ataxia telangiectasia Hyper IgM syndrome Transcobalamin II deficiency and hypogammaglobulinemia X-linked agammaglobulinemia (XLA) (Bruton’s disease) X-linked lymphoproliferative disease associated with EBV infection	Human immunodeficiency virus (HIV) Congenital rubella, Cytomegalovirus (CMV), Ebstein-Barr virus (EBV) Toxoplasma gondii	Chronic lymphocytic leukemia Good syndrome (immunodeficiency with thymoma) Non-Hodgkin’s/Hodgkin’s lymphoma Monoclonal gammopathy MGUS (monoclonal gammopathy of unknown significance)

## Data Availability

The clinical data presented in this case report are not publicly available due to privacy and ethical restrictions. All other data supporting the findings of this study are derived from previously published literature and are available in the referenced articles.

## Abbreviations

AA	Secondary amyloidosis
ACE	Angiotensin-converting enzyme
ACEIs	ACE inhibitors
AV	Alveolar volume
BALF	Bronchoalveolar lavage fluid
BMI	Body mass index
CAPS	Cryopyrin-associated periodic syndromes
CHF	Chronic heart failure
CKD	Chronic kidney disease
CMV	Cytomegalovirus
COPD	Chronic obstructive pulmonary disease
CRP	C-reactive protein
CT	Computed tomography
CVID	Common variable immunodeficiency
c-ANCA	Cytoplasmic-staining antineutrophil cytoplasmic antibody
DLCO	Diffusing capacity of the lungs for carbon monoxide
ds-DNA	Double-stranded DNA
EBV	Epstein–Barr virus
ESID	European Society of Immune Deficiencies
FEV1	Forced expiratory volume in 1 s
FMF	Familial Mediterranean fever
FVC	Forced vital capacity
GI	Gastrointestinal
GLILD	Granulomatous and lymphocytic interstitial lung disease
HIV	Human immunodeficiency virus
hs-Troponin	High-sensitivity troponin
ICON	International consensus document
ID	Interstitial lung disease
IEI	Inborn errors of immunity
IFE	Immunofixation electrophoresis
IgRT	Treatment with immunoglobulin replacement
ILD	Interstitial lung disease
JMF	Jeffrey Modell foundation
MALT	Mucosa-associated lymphoid tissue
MGUS	Monoclonal gammopathy of unknown significance
MM	Multiple myeloma
N/A	Not available
NKT cells	Natural killer T cells
NT-proBNP	N-terminal pro–B-type natriuretic peptide
NYHA	New York Heart Association
OMIM	Online Mendelian Inheritance in Man
p-ANCA	Perinuclear anti-neutrophil cytoplasmic antibodies
PHT	Pulmonary hypertension
SAA	Serum amyloid A
SPEP	Serum protein electrophoresis
TRAPS	Tumor necrosis factor receptor-associated periodic syndrome
VEXAS	Vacuoles, E1 enzyme, X-linked, autoinflammatory, somatic
WES	Whole-exome sequencing
wtATTR	Wild-type transthyretin amyloidosis
XLA	X-linked agammaglobulinemia
